# Clinical Endocannabinoid Deficiency Reconsidered: Current Research Supports the Theory in Migraine, Fibromyalgia, Irritable Bowel, and Other Treatment-Resistant Syndromes

**DOI:** 10.1089/can.2016.0009

**Published:** 2016-07-01

**Authors:** Ethan B. Russo

**Affiliations:** PHYTECS, Vashon Island, Washington.

**Keywords:** anandamide, anorexia nervosa, cannabidiol, cannabinoids, depression, endocannabinoids, fibromyalgia, Huntington disease, irritable bowel syndrome, migraine, motion sickness, multiple sclerosis, Parkinson disease, post-traumatic stress disorder, prebiotics, THC

## Abstract

Medicine continues to struggle in its approaches to numerous common subjective pain syndromes that lack objective signs and remain treatment resistant. Foremost among these are migraine, fibromyalgia, and irritable bowel syndrome, disorders that may overlap in their affected populations and whose sufferers have all endured the stigma of a psychosomatic label, as well as the failure of endless pharmacotherapeutic interventions with substandard benefit. The commonality in symptomatology in these conditions displaying hyperalgesia and central sensitization with possible common underlying pathophysiology suggests that a clinical endocannabinoid deficiency might characterize their origin. Its base hypothesis is that all humans have an underlying endocannabinoid tone that is a reflection of levels of the endocannabinoids, anandamide (arachidonylethanolamide), and 2-arachidonoylglycerol, their production, metabolism, and the relative abundance and state of cannabinoid receptors. Its theory is that in certain conditions, whether congenital or acquired, endocannabinoid tone becomes deficient and productive of pathophysiological syndromes. When first proposed in 2001 and subsequently, this theory was based on genetic overlap and comorbidity, patterns of symptomatology that could be mediated by the endocannabinoid system (ECS), and the fact that exogenous cannabinoid treatment frequently provided symptomatic benefit. However, objective proof and formal clinical trial data were lacking. Currently, however, statistically significant differences in cerebrospinal fluid anandamide levels have been documented in migraineurs, and advanced imaging studies have demonstrated ECS hypofunction in post-traumatic stress disorder. Additional studies have provided a firmer foundation for the theory, while clinical data have also produced evidence for decreased pain, improved sleep, and other benefits to cannabinoid treatment and adjunctive lifestyle approaches affecting the ECS.

## Introduction: Background History and Theory of Clinical Endocannabinoid Deficiency

The theory of clinical endocannabinoid deficiency (CED) was presented in 2001 in two publications,^1,2^ but more thoroughly explored in 2004^3^ in an article that has subsequently been cited frequently in the literature.^4^ The theory of CED was based on the concept that many brain disorders are associated with neurotransmitter deficiencies, affecting acetylcholine in Alzheimer's disease, dopamine in parkinsonian syndromes, serotonin and norepinephrine in depression, and that a comparable deficiency in endocannabinoid levels might be manifest similarly in certain disorders that display predictable clinical features as sequelae of this deficiency.

All humans possess an underlying endocannabinoid tone that reflects of levels of anandamide (AEA) and 2-arachidonoylglycerol (2-AG), the centrally acting endocannabinoids, their synthesis, catabolism, and the relative density of cannabinoid receptors in the brain. If endocannabinoid function were decreased, it follows that a lowered pain threshold would be operative, along with derangements of digestion, mood, and sleep among the almost universal physiological systems subserved by the endocannabinoid system (ECS).^5^ The CED theory also posits that such deficiencies could arise due to genetic or congenital reasons or be acquired due to intercurrent injury or disease that consequently produces characteristic pathophysiological syndromes with particular symptomatology.

**FIG. 1.**
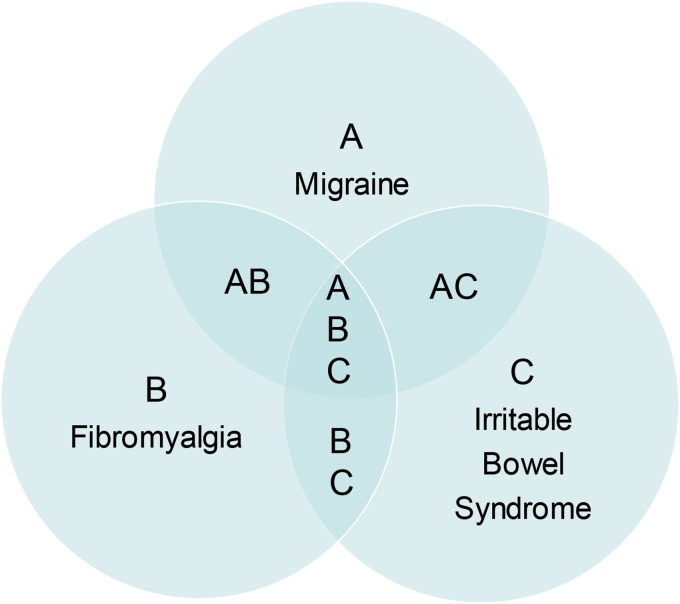
Diagram depicting comorbidity of migraine, fibromyalgia, and irritable bowel syndrome.

The greatest evidence for CED is present for migraine, fibromyalgia, and irritable bowel syndrome (IBS).^3^ A strong case can be advanced for unifying pathophysiological trends in the three conditions:

• All manifest hyperalgesic states must be clinically diagnosed based on subjective criteria as all lack characteristic tissue pathology or easily accessible objective laboratory findings
• All are diagnoses of exclusion that often generate extensive negative diagnostic work-ups
• They display elevated incidence of anxiety and depression (in a chicken vs. egg dilemma) and have been labeled psychosomatic in origin or worse, wastebasket diagnoses, at one time or another by skeptical clinicians
• Comorbidity is quite clear in the three diagnoses. Primary headaches co-occurred in 97% of 201 fibromyalgia patients,^6^ 35.6% of 101 chronic daily headache (transformed migraine) subjects also fit clinical criteria of fibromyalgia,^7^ and 31.6% of IBS subjects were also diagnosable with fibromyalgia, while 32% of fibromyalgia patients also fit for IBS^8^
• While some patients suffer from only one of these syndromes, lifetime risk to develop another or all three is quite common (Fig. 1).

An extensive list of other disorders previously cited that may fall under the CED rubric included^3^ neonatal failure to thrive,^9^ cystic fibrosis,^10^ causalgia,^11^ brachial plexopathy,^12^ phantom limb pain, infantile colic, glaucoma,^13^ dysmenorrhea,^14^ *hyperemesis gravidarum*,^15^ unexplained fetal wastage (repetitive miscarriages), post-traumatic stress disorder (PTSD),^16,17^ bipolar disease,^18^ and possibly many others. All display as yet unfathomed pathophysiological features and remain treatment resistant. Might their underlying nature have been missed? Recently, in a seminal article on the ECS and its optimization,^4^ the authors added support for these and various other conditions.

## Materials and Methods

Standard searches were undertaken of the PubMed/National Library of Medicine database for the listed keywords and references from pertinent literature for pertinence to clinical cannabinoid deficiency.

## IBS, CED, and the Microbiome–Gut–Brain Axis

IBS, also known as spastic colon, is a functional disorder characterized by gastrointestinal (GI) pain, spasm, discomfort, and altered bowel movements, either predominantly diarrhea, predominantly constipation, or alternating between those states. Attacks are highly correlated with anxiety, but debate continues as to which incites the other. Individual episodes may be triggered by some specific foods or dietary indiscretions such as overeating on holidays. While frequently assessed as a life-long condition,^19^ it is clear that significant gastrointestinal insults such as food poisoning or antibiotic administration may generate attacks that persist, often indefinitely. IBS is the most frequent diagnosis in gastroenterology practices in the United States, with prevalence in the Western world of 10–15%.^19^ As an idiopathic disorder, no physical signs are pathognomonic, and even diagnostic procedures such as laboratory tests, including those for gluten enteropathy, colonoscopy, or barium studies, most often fail to identify other causes,^3^ but more formal Rome criteria have been established.^19^ Those authors characterized the status of IBS as (p. 409) a disorder of unknown origin being treated by agents with an unknown mechanism of action. It has been posited that IBS represents a visceral hypersensitivity, with features of GI allodynia and hyperalgesia.^20^ A seminal review of the ECS and its relationship with the GI tract appeared that year.^21^ To summarize, GI propulsion, secretion, and inflammation in the gut are all modulated by the ECS, providing a rationale for cannabinoids as treatment candidates for IBS.^22^ As examples, GI propulsion is under tonic control of the ECS,^21^ and cannabis was one of the first effective clinical interventions in the 19th century for the intense secretory diarrhea associated with cholera,^23^ a finding which was more recently validated with modern methodology.^24^

The use, by its sufferers, of cannabis-based agents to treat IBS has eventuated in large part due to the unfortunate fact that conventional treatment with anticholinergics, opioids, and antidepressants has been quite suboptimal, while three dedicated agents have been withdrawn from certain markets after prior regulatory approval. Two 5-HT_3_ antagonists, alosetron and cilansetron, were associated with ischemic colitis, while tegaserod, a 5-HT_4_ agonist, produced cardiovascular adverse events.

Additional support for the ECS as a key modulator of GI function was provided in an examination of circular muscle fibers from colonoscopic biopsies of surgical specimens from 31 normal patients.^25^ AEA colocalized with cholinergic receptors in normal colon and inhibited the cholinergic contractile force of circular and longitudinal muscles through a non-CB_1_ mechanism or possibly an alternative cannabinoid mechanism not mediated by CB_1_ or CB_2_. It was posited that inflammatory and disease states in the gut rendered the ECS more functionally important.

A 3.5-fold elevation in TRPV1-immunoreactive nerve fibers was observed in biopsies from IBS sufferers compared with controls (*p*<0.0001).^26^ The authors observed (p. 923) that the increased TRPV1 nerve fibers may contribute to visceral hypersensitivity and pain in IBS and provide a novel therapeutic target. Thus, a rationale exists for therapeutic interventions that would boost AEA levels or desensitize TRPV1, such as cannabidiol (CBD), to treat the condition.^27^ Although fatty acid amide hydrolase (FAAH) inhibition of CBD has been questioned by some, its ability to raise serum AEA levels was clearly indicated when administered in high doses to schizophrenic patients.^28^

Genetic variation affecting endocannabinoid metabolism was observed in diarrhea-predominant IBS patients.^29^ THC (dronabinol) treatment slowed colonic transit time in subjects harboring the *CNRI* rs806378 *CT/TT* genotype. Subsequently, a statistically significant association of this gene with colonic transit in IBS with diarrhea (IBS-D) was demonstrated (*p*=0.014).^30^ They observed (p. G559) that CB_1_ receptor-related mechanisms modify colonic transit and sensation and may influence the development of symptoms in Caucasian patients with IBS, particularly IBS-D.

Unfortunately, while many patient surveys have touted benefits of cannabinoid treatment of IBS symptoms^31^ and abundant anecdotal support is evident on the Internet, little actual clinical work has been accomplished. In a randomized controlled trial (RCT) of 52 normal patients taking single doses of 7.5 mg of THC versus placebo, the drug increased colonic compliance (*p*=0.045) and inhibited postprandial colonic tone (*p*=0.048) and fasting and postprandial phasic pressure (*p*=0.008), with a trend toward relaxation of fasting colon tone (*p*=0.096).^32^ Another study focused on visceral sensitivity to rectal distention as measured by a barostat in normal (*N*=12) versus IBS (*N*=10) patients after administration of THC.^33^ No significant differences were noted, but adverse events were reported in 100% of participants at the 10 mg dosage. A third small (23 IBS patients) trial of synthetic THC for a brief interval (2 days) showed no change in transit time.^29^ More formal studies with whole cannabis extracts would be illuminating.

Additional interventions may be practical on the nutritional front utilizing new knowledge of the utility of probiotics and prebiotics. A direct effect of *Lactobacillus acidophilus* NCFM strain through oral administration to induce *CNR2* mRNA expression above that of resting human HT-29 epithelial cells (*p*<0.01) was demonstrated along with an enhancement of morphine antinociceptive effect in rats (*p*<0.001), which was inhibited by administration of the CB_2_ antagonist, AM-630 (*p*<0.001).^34^ A review of human studies of probiotic supplements to treat IBS revealed that 34/42 trials demonstrated beneficial effects for one or more end-points or target symptoms (pain, discomfort, bloating, distention, laboratory parameters).^19^ The interplay of the microbiome–gut–brain axis in IBS is underscored by the recent finding that THC altered the microfloral balance in obese diet-induced obese mice, affecting the Firmicutes:Bacteroidetes ratio (*p*=0.021) and preventing its increase or weight gain despite a high-fat diet.^35^ Thus, optimal gut health without pain and with maintenance of appropriate body weight seems to require a complex interplay between diet, enteric flora, and endocannabinoid balance.

Experimental models have obvious limitations, and contrary findings are always possible. A recent study^36^ demonstrated in a mouse model of accelerated GI transit that palmitoylethanolamide, an entourage endocannabinoid, indirectly activated CB_1_ receptors only under conditions in which AEA or the receptors were upregulated, not deficient. Furthermore, it is unfortunate that laboratory measures of serum or tissue endocannabinoid levels have not been systematically examined in IBS.

## Migraine and CED

Migraine is an extremely prevalent headache syndrome affecting 14% of Americans, with a 3:1 female:male ratio and $20 billion annual cost in that country.^37^ This author has previously reported on migraine's treatment by cannabis,^1,3,38^ and two major reviews have recently appeared.^39,40^ Migraine is far more complex than merely cranial pain. It has a genetic predilection and female predominance and presents as a predominantly hemicranial beating headache associated with unusual associated manifestations: nausea, photophobia, and phonophobia, with hormonal and environmental triggers.

The possible relationship of migraine with the ECS is highlighted by numerous findings. Anandamide produced serotonin receptor responses consisting of 89% potentiation of 5-HT_1A_ and 36% inhibition of 5-HT_2A_,^41^ findings that have been associated with profiles of effective pharmacological migraine interventions that would seem to support respective activity in acute and chronic migraine (CM), respectively. The migraine epiphenomena of photophobia and phonophobia suggest an overactive sensory hyperalgesia, just the kind of homeostatic imbalance that the ECS tends to correct in central nervous system (CNS) function.^5^ The periaqueductal gray matter is a putative migraine generator in which AEA is tonically active, producing analgesia when administered or hyperalgesia when CB_1_ is pharmacologically blocked.^42^

A great deal of additional support for the integral role of the ECS in migraine pathophysiology has been provided by a series of investigations linking endocannabinoids to the trigeminovascular system, which many consider to lie at the root of its pathophysiology. The first experiment^43^ resulted in several pertinent findings: AEA diminished blood vessel dilation in the dura mater induced by calcitonin gene-related peptide (CGRP) 30%, capsaicin 45%, and nitric oxide (NO) 40%. Additionally, AEA acted presynaptically to prevent release of NO by CGRP in dural artery smooth muscle. AEA also was released in tonic manner and displayed modulatory activity in the trigeminovascular system.

A subsequent article focused on vascular phenomena associated with migraine.^44^ AEA caused dose-dependent dural vessel dilation that was diminished by capsazepine, a TRPV1 antagonist, and by CGRP_8–37_, a CGRP antagonist. (While the vascular effects of this and the prior study may appear contradictory, it should be noted that migraine produces vasoconstriction or vasodilation in different phases and that these are epiphenomena of the disorder, rather than its etiology.) The concentration of AEA that produced these findings was far higher than that required to activate CB_1_. This suggests the possibility that repetitive administration with a TRPV1 agonist such as CBD^27^ could conceivably desensitize the receptor and thus alleviate these pathophysiological mechanisms, much as capsaicin has successfully reduced peripheral neuropathic pain with regular cutaneous administration. Capsaicin has even been utilized intranasally as an acute migraine treatment,^45^ and it is thus reasonable to consider CBD as a less noxious alternative desensitizing intervention.

A third publication examined trigeminovascular neuronal responses^46^ with findings that WIN 55,212-2, a potent CB_1_ agonist, inhibited trigeminocervical complex A and C-fiber afferent activity, which was abrogated by SR141716A, a CB_1_ inverse agonist. However, this finding was only obtained with AEA after prior TRPV1 blockade by capsazepine. These findings support possible clinical application of CB_1_-agonists in migraine and cluster headache, although the authors warned of psychoactive sequelae of agents such as THC.

In an animal model of migraine,^47^ AEA reduced nitroglycerin-induced neuronal activation in the nucleus trigeminalis caudalis and area postrema, the latter being an emetic chemoreceptor. There was likewise an induction of expression of the immediate early gene transcription factor Fos in the hypothalamic paraventricular and supraoptic nuclei, in the parabrachial nucleus, and in the brainstem periaqueductal gray matter of the brainstem. These findings reinforce an important role of the ECS in generation of migraine episodes.

Various studies in Italy have focused on the etiological relationship of platelets with migraine in affected patients. In one^48^ of the studies, increased function in AEA membrane transporter and AEA hydrolase (now known as fatty acid amidohydrolase [FAAH], the enzyme that catabolizes AEA) in platelets of women with migraine without aura was observed in comparison with patients with episodic tension headache or controls with no headaches. Interestingly, there were no differences in CB_1_ receptor density in the groups, but AEA hydrolysis was elevated in platelets of migraine sufferers. Consequent decreased serum AEA levels could theoretically lower the pain threshold in such patients.

In another study,^49^ female and male migraineurs both displayed lower FAAH and AEA membrane transporter platelet activity, hypothesized as a possible adaptive response to CM or a reaction to overuse of pain killers known as analgesic rebound. An additional study^50^ showed that 2-AG and AEA levels were both profoundly reduced in the platelets of patients with episodic migraine without aura (*N*=20) and CM (*N*=20) versus controls (*N*=20) (*p*<0.0001).

**FIG. 2.**
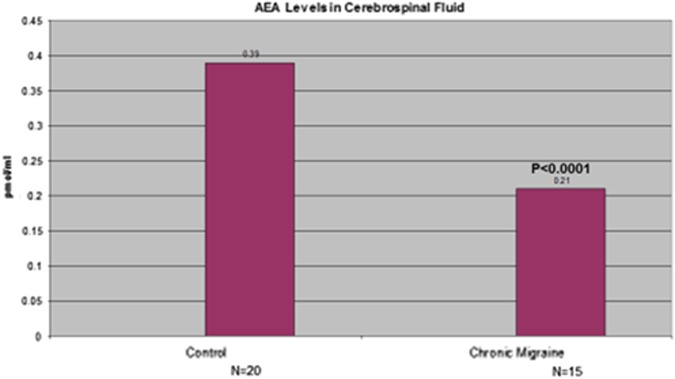
Anandamide levels in cerebrospinal fluid of chronic migraine patients versus controls, adapted from data obtained from Sarchielli et al.^51^

Perhaps the strongest evidence of the existence of CED in migraine or any disorder comes from a study^51^ that assayed cerebrospinal fluid (CSF) AEA levels in 15 chronic migraineurs versus 20 controls with a phenomenal statistically significant difference (*p*<0.0001) (Fig. 2). The authors opined concerning what they termed a system failure in migraine (p. 1387):

Reduced AEA levels in the CSF of CM [chronic migraine] patients support the hypothesis of the failure of this endogenous CB [cannabinoid] system in CM, which seems to be related to increased CGRP and NO production in this pathological condition. This finding might be due to a failure of the inhibitory role of the endocannabinoid AEA on the trigeminovascular system activation—.

THC (1–20 μM) and other CB_1_ agonists dose-dependently diminished cortical spreading depression amplitude, duration, and propagation velocity (*p*<0.001) in a rat brain model, supporting its ability to inhibit the trigeminovascular in migraine with aura.^52^

A clinical study examined 27 medication-overuse headache patients, a common precipitant of migraine exacerbation.^53^ Before treatment, patients displayed decreased temporal summation thresholds, increased pain sensation, and reduced platelet FAAH (the enzyme that breaks down AEA) expression versus controls. After medication withdrawal treatment and elimination of analgesic rebound effects, FAAH activity, and temporal summation thresholds significantly normalized (both *p*=0.001), supporting an etiological ECS dysfunction in these patients.

In subsequent experiments in mice,^54^ intraperitoneal injection of nitroglycerine induced mechanical hyperalgesia that was almost totally eliminated by FAAH deletion or administration of FAAH inhibitors (*p*<0.0001).

Additional supportive data on the migraine-ECS relationship are derived from genetic investigation. The CB_1_ gene, *CNR1* mapped to chromosome 6q14-15, was linked to migraine through haplotypic tagging with high significance (*p*=0.008) and indicative of a genetic effect altering trigeminovascular activation.^55^ The strongest linkage was to HT6 haplotype (*p*=0.002), which correlated highly with migraine symptoms of photophobia > nausea > disability. Migraineurs also showed greater degrees of neuroticism (*p*<0.001), depression (*p*<0.001), and reported drug/alcohol abuse (*p*<0.005). Of late, many pharmaceutical companies have pursued development of antibodies aimed at CGRP as a therapeutic target in migraine prophylaxis,^37^ but it remains to be seen whether this represents a more fundamental target than strategies focusing on the ECS.

**FIG. 3.**
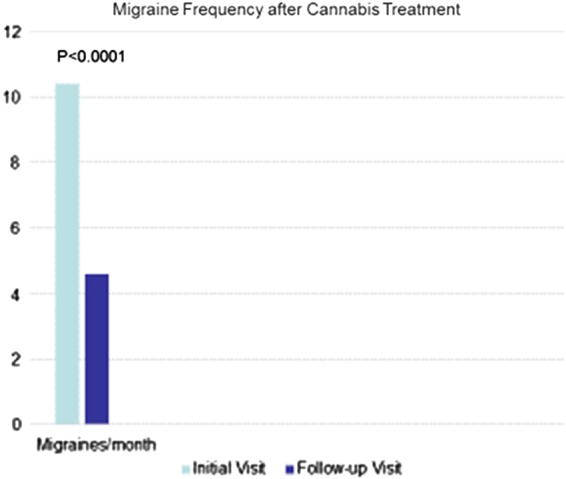
Bar graph of change in migraine frequency after cannabis treatment, adapted from data from Rhyne et al.^57^

Until recently, only case reports and surveys of use of THC and cannabis and its effects on migraine have been published,^31,56^ but a more formal observational trial has been reported^57^ from a cannabis-oriented clinic in the state of Colorado. Among 120 adults with migraine for whom cannabis prophylaxis was recommended, and of which 67.8% had previously used cannabis, the frequency of headache diminished from 10.4 to 4.6 attacks per month (*p*<0.0001) (Fig. 3). Overall, 85.1% had decreased migraine frequency, with 39.7% reporting positive effects: prevention of or reduced headache frequency (19.8%) or aborted headache (11.6%) in this selected and uncontrolled population employing a mixture of administration techniques with unanalyzed but presumably high-THC cannabis.

It is worth remembering that cannabis was a mainstay of treatment of migraine in Europe and North America for a century between 1843 and 1943,^1^ similarly supporting claims of a high degree of efficacy of cannabis treatment in both acute and prophylactic treatments of migraine. Further study utilizing modern techniques and standardized preparations with low THC and higher titers of CBD in proper RCTs is long overdue.

## Focus on Fibromyalgia

Fibromyalgia was probably first described by Sir William Gowers^58^ as fibrositis, a condition characterized as soft tissue pain that could wander in the body, and which was aggravated by overuse. In the 1980s, fibromyalgia became the preferred term due to a failure to identify inflammation or other objective changes in tissue biopsies from affected patients. Formal diagnostic parameters (Rome Criteria) were established thereafter. While a recent report indicated the presence of small fiber neuropathy in a subset of patients with fibromyalgia symptoms^59^ creating possible diagnostic confusion, this finding by no means explains all such cases. Fibromyalgia is noteworthy for its characteristic painful nodules dubbed as trigger points that are particularly prevalent in the shoulder and neck that are frequently of sufficient severity to limit physical activity. The disorder has a clear association with depression and anxiety, but debate surrounds the timing and relationship of these comorbidities. Like migraine, it is more prevalent in women and invariably disrupts sleep. The disorder remains controversial in some quarters, but it is nonetheless the most common diagnosis in American rheumatology practices.^60^ Many authorities now posit a central sensitization consistent with neuropathic pain at the root of the syndrome.^61^ In Italy, it was noted that fibromyalgia, like migraine, was associated with secondary hyperalgesia, that is, a lowered threshold to pain in areas adjacent to the primarily affected parts,^62^ for which the authors suggested pharmacological NMDA blockade for what they interpreted as a deficit in serotonergic analgesia. That same year, hyperalgesia was observed in association with central endocannabinoid hypofunction in the spinal cord and that endocannabinoids reduced associated hyperalgesia,^63^ making the ECS a prime target and CED a rational explanation. The authors proposed that cannabinoid treatments would be indicated for various maladies driven by a primary afferent barrage, which would include visceral hyperalgesia (as hypothesized in IBS), allodynia associated with neuropathic pain states, and reflex sympathetic dystrophy or complex regional pain syndrome.

**FIG. 4.**
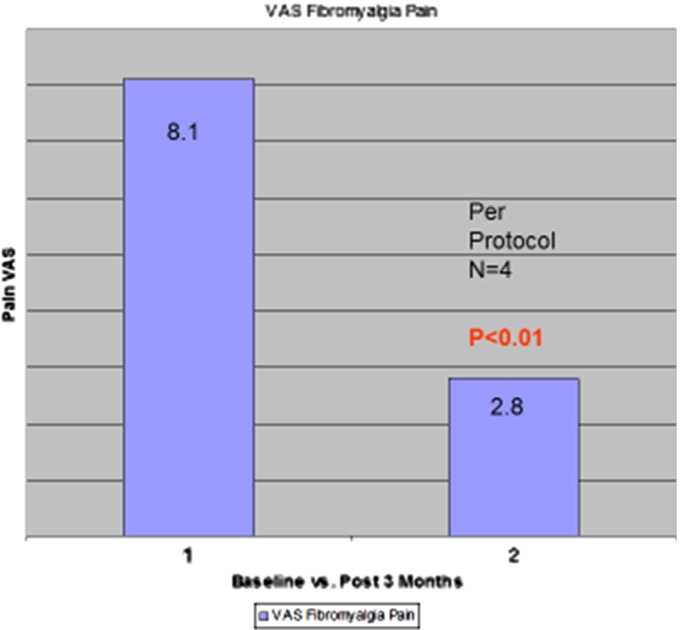
Bar graph depicting decreases in pain in the per-protocol subset of fibromyalgia patients taking THC, adapted from data from Schley et al.^64^ THC, tetrahydrocannabinol (dronabinol).

Cannabis or cannabinoids have been frequently utilized by fibromyalgia patients to treat its myriad symptoms. In an uncontrolled trial in nine patients, THC was administered in doses of 2.5–15 mg a day for 3 months.^64^ Surprisingly, the ethics committee would not permit placebo use in the study. Unfortunately, all but four patients left the study early secondary to THC side effects, but those completing had marked reductions in subjective pain visual analog scales (VAS) (*p*<0.01) (Fig. 4). No benefits on touch-evoked allodynia, nor pinprick hyperalgesia, were documented.

Another group examined nabilone, a semisynthetic THC analog and CB_1_ agonist of 10-fold higher potency.^65^ Forty fibromyalgia patients received nabilone 1 mg BID for 4 weeks. Visual analog scales of pain, a Fibromyalgia Impact Questionnaire, and anxiety scores were all statistically significantly benefited compared with placebo (*p*<0.02). The effects on sleep were also assessed with nabilone^66^ in 31 patients with doses of 0.5–1 mg at bedtime compared with patients taking amitriptyline 10–20 mg. Nabilone was superior on an Insomnia Severity Index, but no benefits on pain, measure of mood, or quality of life were observed.

Herbal cannabis was utilized in an open-label manner in 28 fibromyalgia patients in comparison with an equal number of matched control patients^67^ in another report. Two hours after cannabis use, VAS scores showed a statistically significant (*p*<0.001) reduction of pain and stiffness, enhancement of relaxation, and an increase in somnolence and feeling of well-being. The mental health component summary score of the Short Form (36) Health Survey (SF-36) was significantly higher (*p*<0.05) in cannabis users than in nonusers.

Other cannabis-based medicine clinical trials have been noteworthy in their benefits on symptomatic reduction allowing sleep (reviewed in Refs.^68,69^), and the same would likely be obtained in fibromyalgia, which displays many features in common with other causes of peripheral neuropathic pain. A notable example would be adjunctive use of Sativex (USAN: nabiximols) in a 5-week RCT in 125 patients with intractable peripheral neuropathic pain with allodynia in which it proved superior to placebo (*p*=0.00) in Box Scale-11 (BS-11) score change and reduced dynamic allodynia test scores versus placebo (*p*=0.0420).^70^

While this degree of benefit is yet to be shown in formal RCTs in fibromyalgia, the court of public opinion supports its utility. A recent survey on efficacy of three regulatory body-approved pharmaceutical fibromyalgia treatments versus cannabis recently garnered in excess of 1300 respondents and is available online from the National Pain Report.^71^

**FIG. 5.**
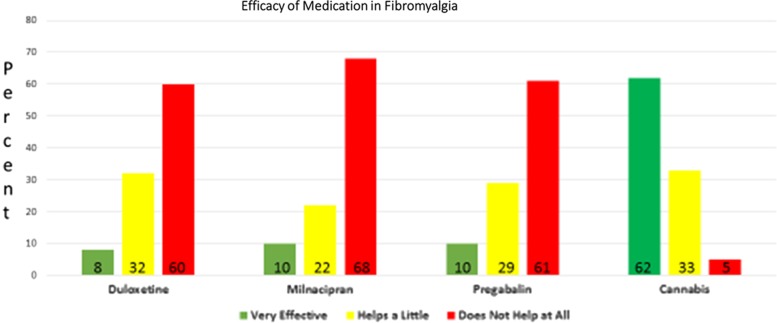
Efficacy of approved pharmaceuticals compared with cannabis in fibromyalgia according to patient survey results, adapted from data from National Pain Report.^71^

Of the approved drugs for fibromyalgia, duloxetine and milnacipran are mixed serotonin and adrenergic uptake inhibitors, while pregabalin is an anticonvulsant drug repurposed to treat neuropathic pain. Results of the survey (Fig. 5) strongly favor cannabis over the poorly effective prescription medicines. These results certainly support an urgent need for more definitive RCTs of a well-formulated and standardized cannabis-based medicine in fibromyalgia inasmuch as existing current medicines with regulatory approval seem to fall quite short of the mark.

## Additional Conditions Suggesting CED

The ECS has been demonstrated to play a key role in the pathophysiology of motion sickness^72^ assessed by subjecting volunteers to parabolic flight maneuvers producing microgravity. Seven of 21 adults so tested developed acute motion sickness with significant reductions in AEA (*p*=0.04) and 2-AG (*p*=0.01) in blood. Nausea scores correlated negatively with AEA (*p*=0.02), and even CB_1_ receptor mRNA gene expression in leukocytes diminished significantly (*p*=0.03) 4 h after exposure in the most adversely affected.

Animal models have clearly established the role of the ECS in multiple sclerosis (MS).^73^ Direct assays of AEA and 2-AG in the CSF of MS patients versus controls confirm significant deficits in affected patients, particularly in secondary progressive cases, confirming an impaired endocannabinoid system,^74^ and affirming the value of such measurements as a functional disease marker.

In a recent interesting finding assessing central pain mechanisms in neuropathy due to diabetes, streptozotocin was administered in a rat model that demonstrated reduced rostroventromedial medullary AEA levels, and in which the TRPV1 desensitizer, capsaicin, decreased nociceptive behavioral signs,^75^ as well as demonstrating the central effects of a supposedly peripheral disorder. If corroborated in human studies, this finding might add diabetic neuropathy to the growing list of putative endocannabinoid deficiency disorders.

In 2008, a mouse model of Huntington's disease (HD) demonstrated a widespread impairment of endocannabinoid function.^76^ Subsequently, in the postmortem brains of human patients with HD,^77^ a striking loss of immunoreactivity of CB_1_ in putamen and globus pallidus was demonstrated throughout the time course of the disorder. The degree of change was much higher than that for enkephalin or substance P, making CB_1_ a superior marker, even at earlier clinical stages. This loss of CB_1_ was felt to be a potential compensatory response as it could reduce GABA release in the striatum. A subsequent positron emission tomography (PET) study in living HD sufferers,^78^ employing 18F-MK9470, a CB_1_ ligand, demonstrated significant decreases in receptor availability versus controls (*p*<0.0001). These reductions ranged from 15% in cerebellum up to 25% in frontal cortex, confirming underactivity of the ECS in HD that would disrupt neurotransmission and correlated inversely with disease severity.

Direct laboratory measurements were also performed in untreated Parkinson's disease (PD) patients, examining CSF,^79^ and demonstrated a doubling of AEA levels over age-matched controls (*p*<0.001), irrespective of disease stage. The authors posited this as a compensatory mechanism in the striatum of PD patients in an effort to alleviate dopamine depletion. Subsequently, another study^80^ was the first to demonstrate the role of the ECS in synaptic long-term depression in motor circuits in PD. The motor deficits present in rodents with dopamine lesions were reversed by combining a D2 agonist with an endocannabinoid reuptake inhibitor. This finding suggests that progressive dopamine loss in PD in striatal circuits may decrease endocannabinoid tone and that the elevations in anandamide in PD patients may be an attempt to compensate for this loss.

Prior animal research has elucidated the relationship between the ECS, extinction of aversive memories,^16^ and stress-induced analgesia.^17^ This has been supplemented by additional evidence that stress-induced anxiety is directly related to central anandamide deficiency in mice.^81^

One genetic study in humans has linked genetic variants of CNR1, the CB_1_ receptor gene, to fear extinction mechanisms.^82^ Homozygote and heterozygote G-allele carriers of the gene rs2180619 showed prominent extinction of fear in a virtual reality experiment, while A/A homozygotes displayed an absence of fear-potentiated startle reactions, confirming the role of the ECS in human fear extinction.

Recent research in humans has clarified the role of the ECS in post-traumatic stress. Forty-six survivors of the World Trade Center attacks were studied.^83^ Serum 2-AG was significantly reduced in PTSD victims versus those without PTSD symptoms, especially those with direct exposure, suggesting a promotion of retention of aversive memories. A negative relationship was also noted between AEA levels and intrusive symptoms. The authors indicated that research to date suggests a good correlation of lower serum AEA levels to increased CB_1_ receptor binding sites in CNS, as was demonstrated in a PET study of untreated PTSD patients.^84^ The CB_1_-selective radioligand [^11^C]OMAR on PET revealed higher volume of distribution (V_T_) with lower AEA tone in PTSD (*p*=0.001) by 19.5% over healthy controls and 14.5% over traumatized patients without PTSD. Cortisol levels were lower in PTSD and trauma patients versus controls and OMAR V_T_, AEA, and cortisol together correctly identified 85% of PTSD cases. Women had greater CB_1_ receptor availability under basal conditions, suggesting greater susceptibility to development of PTSD, in accord with epidemiological observations. Agents increasing AEA availability were suggested as possible therapy and such availability might reflect compensatory upregulation as a reaction to reduced endocannabinoid levels. Three excellent recent reviews reinforce these findings.^85–87^

The criticality of ECS function in other psychiatric syndromes has been evidenced in studies of major depression, which is now thought of less as a failure of monoamine neurotransmission and more as a disorder of CNS plasticity with an inflammatory component, or even as a degenerative disease^88^ directly linked to endocannabinoid deficiency. Additionally, AEA levels were eightfold higher in CSF of untreated acute schizophrenics than in controls (*p*=0.000), and AEA was negatively correlated with psychotic symptoms (*p*=0.001), representing a compensatory mechanism to the disorder.^89^ Recent clinical trial work supports the utility of cannabidiol in its treatment.^28^

PET was also employed in a study of adult female anorexia nervosa and bulimia patients,^90^ demonstrating that global CB_1_ receptor availability was increased in anorexia over controls in cortical and subcortical areas (*p*=0.0003), in the insula in both anorexia and bulimia patients (*p*=0.01 and *p*=0.004, respectively), and in the inferior frontal and temporal areas in anorexia (*p*=0.02). The authors related these chronic upregulations of CB_1_ activity to presumed ECS hypoactivity (p. 780). Interestingly, peripheral serum AEA is elevated in anorexia. Long ago, a single RCT was undertaken in anorexia nervosa in 11 female patients comparing THC to diazepam in a double-blind crossover study.^91^ No increased weight gain was noted in the THC group, but dosing was seemingly excessive (up to 30 mg daily), as evidenced by paranoid ideation and loss of control in three patients (27%). More recent experience would suggest that lower THC dosing with a cannabis-based preparation, as opposed to pure THC, might yield different results with prospects for not only fewer adverse events, but increased efficacy as well.^92–94^ Certainly, additional trials are warranted in this common and difficult clinical context.

Given the current seemingly increased incidence and recognition of autistic spectrum disorders, it is useful to note their possible relationship with the ECS. Genes associated with these disorders also regulate ECS function: neuroligin-3 R451C-knockin and neuroligin-3 knockout mutations in mice impaired tonic endocannabinoid signaling,^95^ with the authors suggesting therapeutic approaches in the human affliction to address this finding. Similarly, presynaptic β-neurexins controlled synaptic signals in excitatory synapses through regulation of postsynaptic 2-AG production^96^ and were said to be essential for control of tonic endocannabinoid signaling.

## Conclusions, Caveats, and Suggestions for Additional Research on and Treatment of CED

The current review has examined the concept of CED and presented more than a decade of supportive objective evidence. However, certain caveats are necessary. One is that contradictory findings are not only possible but also common. This is due, in part, to the often reciprocal relationships between the two major endocannabinoids, AEA and 2-AG, as expansively demonstrated in a current review^87^: Anandamide is most often the tonic signaling agent of the ECS and regulator of synaptic transmission, while 2-arachidonoylglycerol acts as a phasic signal activator in neuronal depolarization and mediator of synaptic plasticity. Thus, discordant levels of the two endocannabinoids may frequently be encountered. Additionally, while CED may be harmful, excesses clearly are, as well, with obvious examples of obesity, metabolic syndrome, and hepatic fibrosis.^97^

Aside from the evidence of depressed AEA levels in the CSF of migraine sufferers^51^ and the other examples presented here, there has been little direct objective evidence of the CED theory in patients until quite recently. Additional investigations in a similar vein to assess endocannabinoid levels in the serum or spinal fluid of migraine, IBS, and fibromyalgia versus controls would be illuminating. Anatomic and physiological scanning techniques (e.g., fMRI, PET) are not yet capable of producing real-time direct assessments of endocannabinoid levels in living patients, but hopefully research will soon allow this type of screening assessment in health and disease. Similarly, genomic testing has produced great strides in elucidating the mutations responsible for many congenital conditions, but has not yet fully plumbed the depths of regulation of gene function that may well underlie the putative CED conditions discussed herein.

RCTs of CED conditions are certainly well justified on the basis of current data and should replace the current largely uncontrolled black market experiments that desperate patients with these afflictions are contemporaneously forced to undertake in their quest for relief of their symptoms.

Various strategies to treat CED conditions are possible. A direct approach with CB_1_ agonists must recognize the fact that the ECS operates as a homeostatic regulator that sometimes requires a gentle pharmacological nudge, rather than a forceful shove, by synthetic full agonists. Thus, small doses of a weak partial agonist (e.g., THC) should be considered, which would not induce tolerance and may jump-start the ECS. Even THC alone is poorly tolerated or appreciated by patients,^98^ and standardized whole cannabis extracts that contain additional synergistic and buffering components, such as CBD and cannabis terpenoids, are certainly preferable.^93^ Alternatively, FAAH inhibitors will also raise AEA levels, but only CBD among them has achieved current legal commercial market availability. Pharmaceutical approaches affecting endocannabinoid transport or its genetic regulation would also hold promise. Beyond drug interventions, a growing body of knowledge supports the realistic goal that lifestyle approaches should be integral to the treatment of CED; specifically, low-impact aerobic regimens have demonstrated beneficial effects on endocannabinoid function,^99^ and as discussed above, dietary manipulations with probiotics and prebiotics may ameliorate not only IBS symptoms but also the entire spectrum of CED conditions. Ultimately, multimodality approaches are most likely to be fruitful in treatment of these common yet difficult clinical challenges.

## Abbreviations Used

2-AG: 2-arachidonoylglycerol
5-HT: 5-hydroxytryptamine (serotonin)
AEA: arachidonylethanolamide (anandamide)
BS-11: Box Scale-11
CB: cannabinoid
CB_1_/CB_2_: cannabinoid receptor 1 or 2
CBD: cannabidiol
CED: clinical endocannabinoid deficiency
CGRP: calcitonin gene-related peptide
CM: chronic migraine
CNS: central nervous system
CSF: cerebrospinal fluid
ECS: endocannabinoid system
FAAH: fatty acid amide hydrolase
fMRI: functional magnetic resonance imaging
GABA: gamma-aminobutyric acid
GI: gastrointestinal
HD: Huntington's disease
IBS: irritable bowel syndrome
IBS-D: irritable bowel syndrome with diarrhea
MS: multiple sclerosis
NMDA: *N*-methyl-d-aspartate
NO: nitric oxide
PD: Parkinson's disease
PET: positron emission tomography
PTSD: post-traumatic stress disorder
RCT: randomized controlled trial
SF-36: Short Form (36) Health Survey
THC: tetrahydrocannabinol (dronabinol)
TRPV: transient receptor potential vanilloid receptor
VAS: visual analog scale
